# Chemical Composition and Biological Activities of the Essential Oils from Three *Melaleuca* Species Grown in Tunisia

**DOI:** 10.3390/ijms131216580

**Published:** 2012-12-05

**Authors:** Ismail Amri, Emilia Mancini, Laura De Martino, Aurelio Marandino, Hamrouni Lamia, Hanana Mohsen, Jamoussi Bassem, Mariarosa Scognamiglio, Ernesto Reverchon, Vincenzo De Feo

**Affiliations:** 1 Laboratory for Forest Ecology, National Institute for Research in Rural Engineering, Water and Forests, BP 10, 2080 Ariana, Tunisie; E-Mails: amri_amri@live.fr (I.A.); hamrounilam@yahoo.fr (H.L.); 2 Department of Pharmaceutical and Biomedical Sciences, University of Salerno, Via Ponte Don Melillo, 84084 Fisciano (Salerno), Italy; E-Mails: emancini@unisa.it (E.M.); ldemartino@unisa.it (L.M.); aureliomarandino@libero.it (A.M.); 3 Plant Molecular Physiology Laboratory, Center of Biotechnology of Borj-Cèdria, BP 901, 2050 Hammam-Lif, Tunisie; E-Mail: punto80@yahoo.com; 4 Chemistry Laboratory, Higher Institute of Education and Continuous Training, 43 Rue de la Liberté, 2019 Le Bardo, Tunisie; E-Mail: jamoussi_bassem@yahoo.fr; 5 Department of Industrial Engineering, University of Salerno, via Ponte Don Melillo, 84084 Fisciano (Salerno), Italy; E-Mails: mrscogna@unisa.it (M.S.); ereverchon@unisa.it (E.R.)

**Keywords:** *Melaleuca acuminata*, *Melaleuca armillaris*, *Melaleuca styphelioides*, phytotoxic activity, antimicrobial activity

## Abstract

The chemical composition of the essential oils of *Melaleuca armillaris* Sm., *Melaleuca styphelioides* Sm. and *Melaleuca acuminata* F. Muell., collected in Tunisia, was studied by means of GC and GC-MS analysis. In all, 46 compounds were identified, 38 for *M. armillari*s, 20 for *M. acuminata* and eight for *M. styphelioides*, respectively. The presence of a sesquiterpenic fraction (52.2%) characterized the oil from *M. armillaris; M. sthypheliodes* oil was rich in methyl eugenol, a phenolic compound (91.1%), while *M. acuminata* oil is mainly constituted by oxygenated monoterpenoids (95.6%). The essential oils were evaluated for their *in vitro* potentially phytotoxic activity against germination and initial radicle growth of *Raphanus sativus* L., *Lepidium sativum* L., *Sinapis arvensis* L., *Triticum durum* L. and *Phalaris canariensis* L. seeds. The radicle elongation of five seeds was inhibited at the highest doses tested, while germination of all seeds was not affected. Moreover, the essential oils showed low antimicrobial activity against eight selected microorganisms.

## 1. Introduction

The genus *Melaleuca* (family Myrtaceae, subfamily Leptospermoideae) consists of about 230 species rich in volatile oils. Several studies demonstrated the efficacy of some *Melaleuca* essential oils against different types of bacteria and fungi [1–4], as in the case of *Melaleuca alternifolia* essential oil against methicillin-resistant *Staphylococcus aureus* (MRSA), *Legionella pneumophila*, *and Staphylococcus aureus*[5].

Essential oils are becoming more popular, because many synthetic drugs are connected with unpleasant side effects, such as nephrotoxicity or ototoxicity. Volatile oils also represent an interesting alternative due to an emerging resistance of microorganisms against synthetic agents. Essential oils can exert not only bacteriostatic and bactericidal effects, but also demonstrate activity against fungi and yeasts [5].

Moreover, in the past, it seemed that certain *Melaleuca* species may have allelopathic properties, resulting in an inhibition of other species in the same ecosystem [6]. The bare ground in *Melaleuca* forests was reported as an example of allelopathy in this genus [7].

*Melaleuca armillaris* Sm. is one of the most widely cultivated melaleucas. It is commonly known as Bracelet Honey Myrtle and grows into a large spreading shrub or small tree. Literature reports about *M. armillaris* remain scarce. GC/MS investigations of its essential oil revealed the presence of 1,8-cineole as the main component [8–12]. Only little information could be found in the literature about the composition of *Melaleuca styphelioides* Sm. oils. Farag and coworkers [12] reported that the essential oil of this species contained mainly caryophyllene oxide (43.8%) and (−)-spathulenol (9.7%). Previously, the same authors [13] reported that the essential oil of *M. styphelioides* contained mainly caryophyllene (49.9%) and methyl eugenol (26.6%). Only a few reports are available about *Melaleuca acuminata* F. Muell. [14,15].

In continuation of our studies on the possible phytotoxic and antimicrobial activity of essential oils from plants collected in the Mediterranean area [16,17], we studied the chemical composition of the essential oils from *Melaleuca armillaris*, *M. acuminata* and *M. styphelioides* and their possible *in vitro* effects against germination and initial radicle elongation of *Raphanus sativus* L. (radish), *Lepidium sativum* L. (garden cress), *Sinapis arvensis* L. (wild mustard), *Triticum durum* L. (wheat) and *Phalaris canariensis* L. (canary grass) and the antimicrobial activity against eight selected microorganism.

## 2. Results and Discussion

### 2.1. Chemical Composition of the Essential Oils

Hydrodistillation yielded 0.65%, 0.53% and 0.35% of essential oil (on a dry mass basis) for *M. armillaris*, *M. acuminata* and *M. stypheliodes*, respectively. Table 1 shows the chemical composition of the three *Melaleuca* oils; compounds are listed according to their elution order on a HP-5MS column. In all, 46 compounds were identified, 38 for *M. armillaris,* accounting for 99.3% of the total oil, 20 for *M. acuminata*, accounting for 99.7% of the total oil, and eight for *M. styphelioides* (92.4%), respectively.

In the oil from *M. armillaris*, the oxygenated monoterpenoids amounted to 44.6%; on the other hand, the total sesquiterpenic fraction amounted to 51.6% of the total oil. The main compounds are the sesquiterpene *cis-*calamenene (19.0%) and the oxygenated sesquiterpene torreyol (15.1%). Other compounds, in a lesser amount, are dihydrocarveol (9.0%) and α-terpineol (7.7%), both oxygenated monoterpenoids. In the literature, Chabir and coworkers [8,9] reported 1,8-cineole (85.8%) as the most abundant compound in *M. armillaris.* The major compound in the essential oil of *M. styphelioides* was methyl eugenol (91.1%), a phenolic compound. In the literature, Farag and coworkers [12] reported that the essential oil of this species contained mainly caryophyllene oxide (43.8%), followed by (−)-spathulenol (9.7%). In another paper [13], the same authors reported that the essential oil of this species contained mainly caryophyllene (50.0%) and methyl eugenol (26.6%).

In the oil from *M. acuminata,* the oxygenated monoterpenoids amounted to 95.6%, with a total sesquiterpenes amount of 1.7% (0.6% sesquiterpene hydrocarbons and 1.1% of oxygenated sesquiterpenes) of the total oil. *trans*-Pinocarveol (25.1%), dihydrocarveol (23.6%), myrtenol (12.3%) and 1,8-cineole (11.7%) were the most abundant among the oxygenated monoterpenes. In the literature, only two references reported the chemical composition of the essential oil of *M. acuminata*: Smith and coworkers [14,15] reported that cineole was the main component of the essential oil of *M. acuminata*.

### 2.2. Phytotoxic Activity

The three essential oils were evaluated for their phytotoxic activity against germination and radicle elongation (Table 2) of radishes and garden cress, two species frequently utilized in biological assays, and of wild mustard, wheat and canary grass, three weed species.

The oils seem to be ineffective against germination, but they affected the radicle elongation of the five tested seeds. The essential oil of *M. styphelioides,* at all doses tested, significantly inhibited the radicle elongation of garden cress. The radicle elongation of wild mustard and radish were inhibited by *M. acuminata* oil at the highest doses (2.5 μg/mL, 1.25 μg/mL) used. At doses of 1.25 and 0.625 μg/mL, the essential oil of *M. acuminata* significantly inhibited the radicle elongation of canary grass (Table 2). The difference in biological activity of the oils could be attributed to their different chemical composition.

On the other hand, the oil of *M. acuminata* was rich in oxygenated monoterpenoids, *trans*-pinocarveol, dihydrocarveol, myrtenol and 1,8-cineole. *trans*-Pinocarveol was reported as one of the main components of the strong phytotoxic oil from a *Cistus ladanifer* L. population [18].

Yatagai and coworkers [19] reported that the leaf oil of *Melelauca bracteata* F. Muell. had the strongest germination and growth-inhibition activity against radish seeds.

The roots were probably more sensitive than shoots to the phytotoxic activity of the oil; the process of germination was active while the oil probably affected the elongation process. Such activity of the essential oils could help to explain the ecological role of the genus *Melaleuca* in the Mediterranean area.

### 2.3. Antimicrobial Activity

The Minimum Inhibitory Concentration (MIC) and the Minimum Bactericidal Concentration (MBC) values of the essential oils against eight selected microorganisms are reported in Table 3.

The essential oils showed inhibitory activity against the gram-positive pathogens, among which is *S. epidermidis*. Among gram-negative bacteria, *E. coli* was affected by the oil of *M. armillaris* and *M. acuminata.* The essential oil of *M. acuminata* was more active than other oils and presumably this activity is related to the high amounts of oxygenated monoterpenoids.

Farag and coworkers [12,13] reported the effect of the essential oil from the leaves of *M. armillaris*, *M. ericifolia* (Smith), *M. leucadendron* (Linn.) and *M. styphelioides* against the growth of some microorganisms. The results demonstrated that the degree of the microbial inhibition is largely dependent on the species. *M. ericifolia* exhibited the highest inhibitory effects against *Bacillus subtilis* and *Aspergillus niger*. The antimicrobial properties of *Melaleuca* essential oil have been reported in several studies [20] Terpinen-4-ol is considered to be the principal active component of *Melaleuca alternifolia* Cheel (tea tree) oil [21–23]. Terpinen-4-ol could constitute an interesting alternative in the therapy of MRSA infections of the skin [5]. In the literature, it was reported that some members of the Myrtaceae family (*Eucalyptus globulus* and *Melaleuca alternifolia*) whose essential oil consisted mainly of monocyclic and bicyclic monoterpenes (e.g., 1,8-cineole and terpinen-4-ol) effectively inhibited the growth of drug-resistant bacterium strains [5].

## 3. Experimental Section

### 3.1. Plant Material

Leaves of *Melaleuca armillaris*, *M. acuminata* and *M. styphelioides* Sm. were collected from the Botanical Garden of the National Institute of Researches on Rural Engineering, Water and Forests (Ariana, Tunisia) in April 2011. Five samples were collected from more than five different trees, mixed for homogenization and used in three replicates for essential oil extractions. Specimens were identified by Dr. H. Lamia, and voucher specimens were deposited at the herbarium of the Laboratory of Forestry Ecology at the National Institute of Research on Rural Engineering, Water and Forest (Tunisia).

### 3.2. Isolation of the Volatile Oils

One hundred grams of dried leaves of each *Melaleuca* species were ground in a Waring blender and then, subjected to hydrodistillation for 3 h according to the standard procedure described in the *European Pharmacopoeia*[24].

The oils were solubilized in *n*-hexane, dried over anhydrous sodium sulphate and dried under N_2_ to remove hexane. Samples were stored at +4 °C in the dark until tested and analyzed.

### 3.3. GC-FID Analysis

The GC-FID analysis was carried out on a Perkin-Elmer Sigma-115 gas chromatograph equipped with a flame ionization detector (FID) and a data handling processor. The separation was achieved using an apolar HP-5 MS fused-silica capillary column (30 m × 0.25 mm i.d., 0.25 μm film thickness). Column temperature: 40 °C, with 5 min initial hold, and then to 270 °C at 2 °C/min, 270 °C (20 min); injection mode: splitless (1 μL of a 1:1000 *n*-pentane solution). Injector and detector temperatures were 250 °C and 290 °C, respectively. Analysis was also run by using a fused silica HP Innowax polyethylenglycol capillary column (50 m × 0.20 mm i.d., 0.25 μm film thickness). In both cases, helium was used as carrier gas (1.0 mL/min).

### 3.4. GC/MS Analysis

The GC/MS analysis was performed on an Agilent 6,850 Ser. II apparatus, fitted with a fused silica DB-5 capillary column (30 m × 0.25 mm i.d., 0.33 μm film thickness), coupled to an Agilent Mass Selective Detector MSD 5973; ionization energy voltage 70 eV; electron multiplier voltage energy 2000 V. Mass spectra were scanned in the range 40–500 amu, scan time 5 scans/s. Gas chromatographic conditions were as reported in the previous paragraph; transfer line temperature, 295 °C.

### 3.5. Identification of the Essential Oils Components

Most constituents were identified by gas chromatography by comparison of their Kovats retention indices (Ri) with either those of the literature [25,26] or with those of authentic compounds available in our laboratories. The Kovats retention indices were determined in relation to a homologous series of *n*-alkanes (C_10_–C_35_) under the same operating conditions. Further identification was made by comparison of their mass spectra on both columns with either those stored in NIST 02 and Wiley 275 libraries or with mass spectra from the literature [25,27] and a homemade library. The components relative concentrations were obtained by peak area normalization. No response factors were calculated.

### 3.6. Biological Assay

A bioassay based on germination and subsequent radicle growth was used to study the phytotoxic effects of the three essential oils on seeds of *Raphanus sativus* L. cv. “Saxa” (radish), *Lepidium sativum* L. (garden cress) and the three weed species *Sinapis arvensis* L. (wild mustard), *Triticum durum* L. (wheat) and *Phalaris canariensis* L. (canary grass). The seeds of radish and garden cress were purchased from Blumen srl (Piacenza, Italy), while mustard, wheat and canary grass were collected from wild plants. The seeds were surface sterilized in 95% ethanol for 15 s and sown in Petri dishes (Ø = 90 mm) containing five layers of Whatman filter paper impregnated with distilled water (7 mL, control) or a tested solution of the essential oil (7 mL) at the different assayed doses. The germination conditions were 20 ± 1 °C with a natural photoperiod. The essential oils, in a water–acetone mixture (99.5:0.5), were assayed at the doses of 2.5, 1.25, 0.625, 0.25, 0.125 and 0.062 μg/mL. Controls performed with water-acetone mixture alone showed no appreciable differences in comparison with controls in water alone. Seed germination was observed directly in Petri dishes, each 24 h. A seed was considered germinated when the protrusion of the root became evident [28]. After 120 h (on the fifth day), the effects on radicle elongation were measured in centimeter. Each determination was repeated three times, using Petri dishes containing 10 seeds each. Data are expressed as the mean ± SD for both germination and radicle elongation. Data were ordered in homogeneous sets, and the Student’s *t* test of independence was applied [29].

### 3.7. Determination of Minimum Inhibitory Concentration (MIC) and Minimum Bactericidal Concentration (MBC)

The antibacterial activity was evaluated by determining the minimum inhibitory concentration (MIC) and the minimum bactericidal concentration (MBC) using the broth dilution method [30–32]. Eight bacterial species, selected as representative of the gram-positive and gram-negative classes, were tested: *Staphylococcus aureus* (ATTC 25923), *Streptococcus faecalis* (ATTC 29212), *Bacillus subtilis* (ATCC 6633), *Escherichia coli* (ATCC 25922), *Pseudomonas aeruginosa* (ATCC 27853), *Staphylococcus epidermidis* (ATCC 12228), *Klebsiella pneumoniae* (ATCC 10031) and *Proteus vulgaris* (ATCC 13315). The strains were maintained on Tryptone Soya agar (Oxoid, Milan, Italy); for the antimicrobial tests, Tryptone Soya broth (Oxoid, Milan, Italy) was used. In order to facilitate the dispersion of the oil in the aqueous nutrient medium, it was diluted with Tween 20 at a concentration of 10%. Each strain was tested with a sample that was serially diluted in broth to obtain concentrations ranging from 100 μg/mL to 0.8 μg/mL. The sample was previously sterilized with a 0.20 μm Millipore filter. The sample was stirred, inoculated with 50 μL of physiological solution containing 5 × 10^6^ microbial cells and incubated for 24 h at 37 °C. The MIC value was determined as the lowest concentration of the sample that did not permit any visible growth of the tested microorganism after incubation. The control, containing only Tween 20 instead of the essential oil, was not toxic to the microorganisms. Cultures, containing only sterile physiologic solution Tris buffer, were used as positive control. MBC was determined by subculture of the tubes with inhibition in 5 mL of sterile nutrient broth. After incubation at 37 °C, the tubes were observed. When the germs did not grow, the sample denoted a bactericidal action. Oil samples were tested in triplicate and the experiment was performed three times. The results are expressed as mean ± SD. Chloramphenicol was used as reference drug [17].

## 4. Conclusions

Data obtained in this paper could be useful in the chemotaxonomic knowledge of the genus *Melaleuca* that consists of about 230 species, the chemistry of *M. armillaris*, *M. styphelioides* and *M. acuminata* being little known. Data on phytotoxic activities could help to explain the ecological role of genus *Melaleuca* in the Mediterranean area. The antimicrobial activity of the essential oil is in agreement with the uses of other *Melaleuca* species as antimicronial drugs.

## Figures and Tables

**Table 1:** Percent composition of *Melaleuca armillaris*, *Melaleuca acuminata* and *Melaleuca styphelioides* essential oils.

Compound	RI a	RI b	*Melaleuca armillaris*	*Melaleuca acuminata*	*Melaleuca styphelioides*	Identification c
α-Pinene	935	1032	-	-	0.1	1,2,3
Myrcene	989	1162	-	-	t	1,2,3
*p*-Cymene	1017	1269	-	-	0.1	1,2,3
1,8-Cineole	1022	1213	3.6	11.7	-	1,2,3
Perillene	1104	1429	-	0.1	-	1,2
α-Campholenal	1117	1497	-	0.7	-	1,2
Nopinone	1124	1597	0.4	3.0	-	1,2
*trans*-Pinocarveol	1127	1664	6.9	25.1	-	1,2
Camphor	1131	1532	-	1.2	-	1,2,3
1-Terpineol	1133		-	0.5	-	1,2
*trans*-Sabinol	1136	1720	0.6	1.6	-	1,2
*trans*-Verbenol	1139	1683	0.4	1.1	-	1,2
Eucarvone	1150	1932	1.4	6.4	-	1,2
Isoborneol	1155	1633	0.5	0.3	-	1,2
Viridene	1160		1.3	2.4	-	1,2
Terpinen-4-ol	1168	1611	0.4	-	-	1,2
*m*-Cymen-8-ol	1179		0.2	-	-	1,2
*p*-Cymen-8-ol	1180	1856	0.1	-	-	1,2
α-Terpineol	1182	1706	7.7	-	-	1,2,3
Dihydrocarveol	1183	1755	9.0	23.6	-	1,2
Myrtenol	1187	1804	6.4	12.3	-	1,2
Myrtenal	1191	1648	0.8	-	-	1,2
*cis*-Dihydrocarvone	1194	1620	0.5	-	-	1,2
Verbenone	1196	1723	3.7	7.8	-	1,2
*p*-Cymen-9-ol	1200		0.1	0.2	-	1,2
9-Decen-1-ol	1260		0.6	-	-	1,2
Citronellyl acetate	1353	1662	0.5	-	-	1,2
*cis*-Piperitone oxide	1360	1733	0.8	-	-	1,2
Methyl eugenol	1401	2016	-	-	91.1	1,2
4,8-β*-*epoxy*-*Caryophyllane	1424		0.4	-	-	1,2
*cis*-α-Ambrinol	1435		0.6	-	-	1,2
*allo*-Aromadendrene	1446	1661	-	-	0.2	1,2
Aromadendrene	1453	1628	4.1	-	-	1,2
γ-Himachalene	1483		1.5	-	-	1,2
10-Undecen-1-ol, acetate	1495		0.6	-	-	1,2
*cis*-Calamenene	1511		19.0	-	-	1,2
Germacrene B	1539	1854	1.8	0.6	-	1,2
Spathulenol	1562	2150	4.4	0.3	0.4	1,2
Caryophyllene oxide	1565	2008	0.8	0.6	0.4	1,2,3
Globulol	1568	2098	1.2	-	-	1,2
Guaiol	1575	2108	1.3	-	-	1,2
β-Atlantol	1586		0.5	-	0.1	1,2
1-*epi*-Cubenol	1591	2088	0.5	0.2	-	1,2
Torreyol	1615		15.1	-	-	1,2
γ-Eudesmol	1623	2173	0.7	-	-	1,2
Methyl jasmonate	1628		0.6	-	-	1,2
α-Eudesmol	1633	2250	0.3	-	-	1,2
Total compounds			**99.3**	**99.7**	**92.4**	
Monoterpene hydrocarbons			**-**	**-**	**0.2**	
Oxygenated Monoterpenes			**44.6**	**95.6**	**-**	
Sesquiterpene hydrocarbons			**26.4**	**0.6**	**0.2**	
Oxygenated Sesquiterpenes			**25.2**	**1.1**	**0.9**	
Phenolic compound			**-**	**-**	**91.1**	
Carbonylic compounds			**1.2**	**-**	**-**	
Non terpenes			**1.3**	**2.4**	**-**	
Others			**0.6**	**-**	**-**	

a Kovats retention index on HP-5 MS column;

b Kovats retention index on HP Innowax;

c 1 = Kovats retention index, 2 = mass spectrum, 3 = co-injection with authentic compound; t = trace, less than 0.05%.

**Table 2:** Phytotoxic activity of the essential oils of *Melaleuca armillaris*, *Melaleuca styphelioides* and *Melaleuca acuminata* against germination and radicle elongation of *Sinapis arvensis*, *Triticum durum*, *Phalaris canariensis*, *Raphanus sativus* and *Lepidium sativum*, 120 h after sowing. Data are expressed in centimeter.

*Sinapis arvensis* Germinated seeds ± SD (cm)				*Sinapis arvensis* Radicle elongation ± SD (cm)			

Doses	*Melaleuca armillaris*	*Melaleuca styphelioides*	*Melaleuca acuminata*	Doses	*Melaleuca armillaris*	*Melaleuca styphelioides*	*Melaleuca acuminata*
Control	8.8 ± 0.8	8.8 ± 0.8	8.8 ± 0.8	Control	1.4 ± 0.7	1.4 ± 0.7	1.4 ± 0.7
0.062 μg/mL	9.2 ± 0.6	9.2 ± 0.6	9.8 ± 0.6	0.062 μg/mL	1.2 ± 0.7	1.3 ± 0.6	1.9 ± 0.8 **
0.125 μg/mL	8.3 ± 1.4	9.9 ± 0.6	9.4 ± 0.6	0.125 μg/mL	1.3 ± 0.5	1.4 ± 0.6	1.5 ± 0.9
0.25 μg/mL	8.5 ± 1.6	9.2 ± 0.6	9.8 ± 0.6	0.25 μg/mL	1.0 ± 0.4	1.1 ± 0.5	1.4 ± 0.8
0.625 μg/mL	7.9 ± 1.4	9.9 ± 0.6	10.0 ± 0.0 *	0.625 μg/mL	2.3 ± 1.3 *	1.0 ± 0.6	1.1 ± 0.4
1.25 μg/mL	9.2 ± 0.6	9.9 ± 0.6	9.8 ± 0.6	1.25 μg/mL	1.7 ± 1.0	1.3 ± 0.9	1.0 ± 0.6 **
2.5 μg/mL	8.8 ± 1.2	9.5 ± 1.1	9.8 ± 0.6	2.5 μg/mL	1.0 ± 0.4	1.2 ± 0.7	0.9 ± 0.6 **

***Triticum durum*Germinated seeds ± SD (cm)**				***Triticum durum*Radicle elongation ± SD (cm)**			

Doses	*Melaleuca armillaris*	*Melaleuca styphelioides*	*Melaleuca acuminata*	Doses	*Melaleuca armillaris*	*Melaleuca styphelioides*	*Melaleuca acuminata*

Control	8.3 ± 0.9	8.3 ± 0.9	8.3 ± 0.9	Control	4.4 ± 2.3	4.4 ± 2.3	4.4 ± 2.3
0.062 μg/mL	8.7 ± 1.2	9.1 ± 1.3	8.0 ± 1.4	0.062 μg/mL	4.7 ± 2.3	4.7 ± 1.9	4.0 ± 1.7
0.125 μg/mL	8.7 ± 0.6	7.4 ± 3.4	8.6 ± 1.1	0.125 μg/mL	4.5 ± 2.2	4.8 ± 2.2	4.7 ± 1.9
0.25 μg/mL	7.3 ± 1.5	8.0 ± 1.3	7.7 ± 1.1	0.25 μg/mL	5.7 ± 1.6 *	5.3 ± 1.7	4.4 ± 2.6
0.625 μg/mL	8.7 ± 0.6	8.0 ± 2.3	8.0 ± 1.1	0.625 μg/mL	3.2 ± 1.9 *	5.1 ± 1.8	4.2 ± 2.0
1.25 μg/mL	6.7 ± 3.5	9.9 ± 1.1	7.7 ± 0.6	1.25 μg/mL	3.9 ± 1.5	4.9 ± 1.8	2.8 ± 1.7 **
2.5 μg/mL	8.7 ± 1.5	9.6 ± 1.7	8.6 ± 1.1	2.5 μg/mL	3.8 ± 1.7	4.0 ± 1.8	3.4 ± 2.0

***Phalaris canariensis*Germinated seeds ± SD (cm)**				***Phalaris canariensis*Radicle elongation ± SD (cm)**			

Doses	*Melaleuca armillaris*	*Melaleuca styphelioides*	*Melaleuca acuminata*	*Doses*	*Melaleuca armillaris*	*Melaleuca styphelioides*	*Melaleuca acuminata*
Control	8.8 ± 0.9	8.8 ± 0.9	8.8 ± 0.9	Control	3.1 ± 1.2	3.1 ± 1.2	3.1 ± 1.2
0.062 μg/mL	8.8 ± 2.2	9.0 ± 0.6	8.7 ± 1.0	0.062 μg/mL	2.8 ± 1.1	3.2 ± .3	2.8 ± 0.7
0.125 μg/mL	9.2 ± 1.6	9.7 ± 0.0	9.7 ± 0.0	0.125 μg/mL	3.0 ± 1.4	3.3 ± 1.1	3.4 ± 1.2
0.25 μg/mL	9.4 ± 0.6 *	8.1 ± 1.1	8.7 ± 1.0	0.25 μg/mL	2.4 ± 1.2	3.7 ± 1.3	3.2 ± 1.5
0.625 μg/mL	9.2 ± 0.6	8.7 ± 1.0	9.4 ± 0.6	0.625 μg/mL	2.6 ± 1.2	3.9 ± 1.0	2.5 ± 0.9 *
1.25 μg/mL	10.6 ± 0.0 **	8.7 ± 1.7	8.4 ± 1.4	1.25 μg/mL	2.6 ± 0.8	3.2 ± 1.3	1.6 ± 1.4 ***
2.5 μg/mL	9.2 ± 0.6	8.4 ± 0.6	8.1 ± 1.5	2.5 μg/mL	3.5 ± 1.6	2.8 ± 1.1	2.7 ± 0.7

***Raphanus sativus*Germinated seeds ± SD (cm)**				***Raphanus sativus*Radicle elongation ± SD (cm)**			

Doses	*Melaleuca armillaris*	*Melaleuca styphelioides*	*Melaleuca acuminata*	*Doses*	*Melaleuca armillaris*	*Melaleuca styphelioides*	*Melaleuca acuminata*

Control	9.3 ± 0.7	9.3 ± 0.7	9.3 ± 0.7	Control	6.1 ± 2.8	6.1 ± 2.8	6.1 ± 2.8
0.062 μg/mL	10.0 ± 0.0	9.6 ± 0.0	9.0 ± 0.6	0.062 μg/mL	6.7 ± 3.3	6.0 ± 2.5	4.8 ± 2.4
0.125 μg/mL	10.0 ± 0.0	8.9 ± 0.6	10.1 ± 0.6	0.125 μg/mL	6.0 ± 3.4	4.0 ± 2.4 **	5.0 ± 2.2
0.25 μg/mL	10.0 ± 0.0	9.9 ± 0.6	9.4 ± 0.0	0.25 μg/mL	6.4 ± 3.2	5.3 ± 2.4	4.5 ± 2.3
0.625 μg/mL	9.7 ± 0.6	9.6 ± 0.0	9.7 ± 1.1	0.625 μg/mL	7.1 ± 2.9	5.1 ± 2.9	4.4 ± 2.4
1.25 μg/mL	9.7 ± 0.6	8.9 ± 0.6	8.6 ± 1.6	1.25 μg/mL	6.1 ± 2.9	4.0 ± 2.1 **	3.5 ± 2.0 ***
2.5 μg/mL	9.7 ± 0.6	9.3 ± 0.6	9.7 ± 1.6	2.5 μg/mL	6.3 ± 2.9	4.8 ± 2.6	4.2 ± 2.4 *

***Lepidum sativum*Germinated seeds ± SD (cm)**				***Lepidum sativum*Radicle elongation ± SD (cm)**			

Doses	*Melaleuca armillaris*	*Melaleuca styphelioides*	*Melaleuca acuminata*	*Doses*	*Melaleuca armillaris*	*Melaleuca styphelioides*	*Melaleuca acuminata*

Control	8.2 ± 0.5	8.2 ± 0.5	8.2 ± 0.5	Control	2.8 ± 1.8	2.8 ± 1.8	2.8 ± 1.8
0.062 μg/mL	8.2 ± 0.0	8.2 ± 0.6	9.2 ± 1.0	0.062 μg/mL	2.7 ± 1.5	1.5 ± 1.1 ***	2.7 ± 2.1
0.125 μg/mL	8.9 ± 1.5	8.7 ± 0.6	8.2 ± 1.0	0.125 μg/mL	2.5 ± 1.2	1.61.1 ***	2.7 ± 1.5
0.25 μg/mL	9.5 ± 0.6	8.5 ± 1.6	9.2 ± 1.0	0.25 μg/mL	2.3 ± 2.2	1.8 ± 1.4 ***	2.7 ± 2.1
0.625 μg/mL	8.5 ± 0.6	8.5 ± 0.9	9.2 ± 1.0	0.625 μg/mL	2.8 ± 1.4	1.1 ± 1.0 ***	2.7 ± 2.1
1.25 μg/mL	9.2 ± 1.0	7.5 ± 0.9	8.9 ± 1.5	1.25 μg/mL	1.9 ± 1.4	1.8 ± 1.1 **	2.2 ± 1.8
2.5 μg/mL	7.2 ± 1.0	8.7 ± 0.6	7.9 ± 1.5	2.5 μg/mL	2.9 ± 1.4	1.9 ± 1.0 **	2.6 ± 2.4

* *p* < 0.05;

** *p* < 0.01;

*** *p* < 0.001 *vs.* control.

Results are the mean ± standard deviation (SD) of three experiments.

**Table 3:** Minimum Inhibitory Concentration (MIC) and Minimum Bactericidal Concentration (MBC) Values (μg/mL) of essential oils from the three *Melaleuca* Species and MIC of the reference antibiotic, chloramphenicol. Results are the mean of three experiments.

Bacterial strain	*Melaleuca styphelioides*		*Melaleuca armillaris*		*Melaleuca acuminata*		Chloramphenicol
	MIC a	MBC b	MIC	MBC	MIC	MBC	
*Bacillus subtilis* ATCC 6633	100	>100	50	100	50	n.a.	12.5
*Staphylococcus aureus* ATCC 25923	100	>100	100	>100	50	100	25
*Staphylococcus epidermidis* ATCC12228	50	100	50	n.a.	12.5	n.a.	3.12
*Streptococcus faecalis* ATCC 29212	>100	n.a.	>100	n.a.	100	100	25
*Escherichia coli* ATCC 25922	100	>100	50	n.a.	25	50	12.5
*Klebsiella pneumoniae* ATCC 10031	>100	n.a.	100	>100	100	>100	50
*Proteus vulgaris* ATCC 13315	>100	n.a.	50	100	50	100	25
*Pseudomonas aeuriginosa* ATCC 27853	>100	n.a.	>100	n.a.	>100	n.a.	100

a MIC, Minimal Inhibitory Concentration (μg/mL);

b MBC, Minimal Bactericidal Concentration (μg/mL); n.a., not active.
